# Advances in Cytokines and Inflammatory Mechanisms in the Pathogenesis of Interstitial Cystitis/Bladder Pain Syndrome

**DOI:** 10.3390/biom16010138

**Published:** 2026-01-13

**Authors:** Yulin Xiao, Donglin Zhu, Xiangfu Zhou

**Affiliations:** Department of Urology, The Third Affiliated Hospital of Sun Yat-sen University, 600 W Tianhe Rd., Guangzhou 510630, China; xiaoylin5@mail2.sysu.edu.cn (Y.X.); zhudl3@mail.sysu.edu.cn (D.Z.)

**Keywords:** inflammatory mechanisms, cytokines, interstitial cystitis/bladder pain syndrome

## Abstract

Bladder discomfort, urgency, and frequency of urination are the hallmarks of interstitial cystitis/bladder pain syndrome (IC/BPS), a chronic illness. Although the precise etiology remains unclear, numerous clinical investigations have established inflammation as a pivotal factor in its pathogenesis, encompassing uroepithelial damage, mast cell activation, and neuroinflammatory responses. This review delineates the pathological features and classification of IC/BPS, emphasizing the contribution of inflammatory mechanisms and the involvement of cytokines as key mediators in disease progression. The insights presented aim to guide the advancement of innovative treatment approaches.

## 1. Introduction

Interstitial cystitis/bladder pain syndrome (IC/BPS) denotes a range of persistent urological symptoms predominantly marked by pain or pressure in the suprapubic region. These symptoms are often associated with heightened urine frequency, urgency, and/or nocturia, occurring without infection or other identifiable clinical abnormalities. The ailment primarily impacts women aged 40 to 60, with an estimated prevalence of 3 to 8 million females and more than 2 million males diagnosed with bladder pain syndrome in the United States [1]. Furthermore, individuals with IC/BPS exhibit a higher incidence of stress, sleep disturbances, depression, and sexual dysfunction compared to the general population, resulting in a substantially greater impairment in quality of life (QoL) [2]. The fundamental pathophysiological mechanisms of interstitial cystitis/bladder pain syndrome (IC/BPS) are not yet fully elucidated, and its diagnosis is predominantly established through a process of exclusion [3]. A precise diagnosis necessitates a comprehensive clinical history, the presence of sterile urine with negative cytology, cystoscopic hydrodistention performed under anesthesia, and histopathological evaluation of bladder biopsy samples.

At present, several hypotheses have been suggested concerning the pathophysiology of IC/BPS, including epithelial failure, mast cell activation, neurogenic inflammation, autoimmune response, and latent infection [4]. These mechanisms are not mutually exclusive. Rather, they may engage within a multifaceted network that collectively contributes to the initiation and advancement of the disease. Among these factors, the inflammatory response is considered to serve as a fundamental component in the pathological processes underlying IC/BPS. Recent studies increasingly suggest that cytokines are critically involved in the development of IC/BPS. Cytokines, a category of low molecular weight proteins released by immunological and various cell types, modulate cellular proliferation, differentiation, and function by attaching to particular receptors on target cells. In bladder tissues and urine samples from individuals with IC/BPS, significant alterations in the expression levels of various pro- and anti-inflammatory cytokines have been observed [5], implicating these changes in the pathophysiology. This review seeks to systematically clarify the role of inflammation and cytokines in the pathogenesis of interstitial cystitis from a fundamental research standpoint, thereby offering new insights and a theoretical basis for the diagnosis and treatment of IC/BPS.

## 2. Pathologic Features and Classification of IC/BPS

At present, IC/BPS is chiefly classified into two categories based on the occurrence of Hunner’s lesions. Patients diagnosed with interstitial cystitis/bladder pain syndrome exhibiting Hunner’s lesions tend to experience more pronounced central bladder symptoms, fewer concomitant non-bladder comorbidities, and show a more advantageous response to intravesical medication than those lacking Hunner’s lesions [6]. Conversely, IC/BPS patients devoid of Hunner’s lesions frequently present with mental health issues and affective dysregulation [7,8], demonstrating considerable overlap in symptomatology and comorbidities with functional somatic syndromes, including irritable bowel syndrome, fibromyalgia, chronic fatigue syndrome, and migraine headaches [9].

In addition, notable histopathological differences exist between the two subtypes of IC/BPS (Table 1). Interstitial Cystitis/Bladder Pain Syndrome marked by Hunner’s lesions demonstrates specific histological characteristics of the bladder, including epithelial loss and persistent inflammatory alterations, such as infiltration by lymphocytes, plasma cells, and mast cells, together with interstitial fibrosis and edema [10,11]. These lesions typically involve the entire bladder [12]. In contrast, IC/BPS instances devoid of Hunner’s lesions exhibit negligible histologic alterations. The findings suggest that interstitial cystitis/bladder pain syndrome (IC/BPS) characterized by the presence of Hunner’s lesions corresponds to a chronic inflammatory disorder of the bladder. In contrast, IC/BPS cases lacking Hunner’s lesions appear to represent a non-inflammatory condition, devoid of distinct pathological changes in the bladder. Additionally, clinical reports of patients transitioning between these two IC/BPS subtypes are rare [13]. Thus, interstitial cystitis (IC) is typically categorized as IC/BPS with Hunner’s lesions, whereas the subtype lacking Hunner’s lesions is referred to as bladder pain syndrome (BPS).

**Table 1:** Variations in the clinical characteristics of IC/BPS with/without Hunner’s lesions.

	IC/BPS with Hunner’s Lesions	IC/BPS Without Hunner’s Lesions	References
Age at diagnosis	Usually at 60 s	Usually at 40 s	[12,14]
inflammation	Present	Absent or minimal	[11]
Epithelial denudation	Denuded	Preserved	[12]
Infiltrative inflammatory cell type	Lymphocytes and plasma cells predominate	Very low numbers of plasma cells	[15]
Bladder center symptom	Severity	Less severe	[16]
Average bladder capacity	Smaller	Larger	[14]
Interstitial Cystitis Symptom Index (ICSI) scores	Higher	Lower	[17]
Endoscopic management	Effective	Less effective	[18]
Surgical Management	Limited	Effective for urgency and frequency	[18]

## 3. Inflammations and Cytokines in IC/BPS

The initial and early response of the organism to harmful stimulus or injury is characterized by acute inflammation. This process is marked by vasodilation, increased vascular permeability, the migration of leukocytes to the injury site, and the initiation of an inflammatory biochemical cascade resulting in the release of mediators including cytokines, histamine, kinins, complement proteins, coagulation factors, nitric oxide, and proteases [19]. In the context of acute cystitis, these mediators induce congestion and edema of the bladder mucosa, as well as ulcer formation, which predisposes the tissue to hemorrhagic injury. The presence of small, clear vesicles—cystic cavities containing liquid, gaseous, or semi-solid substances—is frequently observed during urinalysis following the stripping of the superficial mucosa. Furthermore, these mediators provoke irritation of the bladder mucosa, manifesting clinically as urinary urgency, frequency, and dysuria [20]. Typically, these inflammatory mediators possess a short half-life and are rapidly degraded, allowing the inflammation to resolve promptly once the harmful stimulus is eliminated. However, if acute inflammation persists, it may progress to subacute inflammation. When inflammation endures beyond six weeks, it transitions into a chronic state, which can result in tissue damage and fibrosis [21], as observed in IC. Chronic inflammation in interstitial cystitis is marked by the infiltration of mononuclear cells, including macrophages, eosinophils, mast cells, lymphocytes, and plasma cells. This cellular infiltration leads to irreversible tissue damage and pathological alterations, like fibrosis, decreased bladder compliance, detrusor overactivity, and increased nociceptive sensitivity [22]. These changes contribute to chronic, recurrent episodes of pain and lower urinary tract symptoms.

Multiple studies have substantiated the involvement of inflammation in interstitial cystitis and have identified the upregulation of various cytokines in serum, urine, and bladder biopsy specimens [23,24,25,26,27]. Ashti et al. [28] employed a rat model of acute interstitial cystitis/bladder pain syndrome generated by cyclophosphamide injection (150 mg/kg). Their dynamic analysis demonstrated that interleukin (IL)-4 was positively correlated with IL-1β, as well as between tumor necrosis factor-alpha (TNF-α) and IL-1β during the initial phase. In the later stages, IL-5, IL-6, and interferon-gamma (IFN-γ) emerged as additional key inflammatory mediators, reflecting the fluctuating severity of inflammation throughout the course of IC/BPS. A prevailing theory [16] postulates that the immune inflammatory response observed in interstitial cystitis is a secondary phenomenon, resulting from the exposure of endogenous pathogens from bladder tissue cells to the immune system. This exposure is typically preceded by cellular damage, metabolic disturbances, or infection-mediated impairment, which subsequently activates an immune response. The persistence of noxious stimuli perpetuates chronic inflammation, which subsequently initiates a series of interconnected cascade responses, thereby establishing a self-reinforcing cycle of prolonged inflammation and repeated injury to the bladder epithelium. Ketamine and its urinary metabolites directly damage the urinary tract epithelium, making ketamine-induced cystitis a viable model for clinically simulating interstitial cystitis [29]. The role of inflammation in the pathogenesis of interstitial cystitis is well-established, with numerous studies identifying inflammatory markers, such as chemokines and cytokines, as biomarkers for this condition. Jiang et al. [30] demonstrated that MCP-1, eotaxin, MIP-1β, TNF-α, and PGE2 could differentiate healthy individuals from IC/BPS patients, while IL-8, CXCL10, brain-derived neurotrophic factor (BDNF), IL-6, and RANTES were effective biomarkers for identifying IC/BPS patients presenting. Niimi et al. further confirmed the diagnostic utility of CXCL10 in interstitial cystitis, particularly among patients with Hunner’s lesions [31]. Another study showed that combining IL-6 with the methylhistamine/creatinine ratio resulted in a sensitivity of 70.0%, specificity of 72.4%, positive predictive value of 77.8%, and negative predictive value of 63.6% for diagnosing interstitial cystitis [32]. Nevertheless, these studies are constrained by small sample sizes. The diagnostic role of cytokines and chemokines in interstitial cystitis warrants further validation through large-scale studies. The clinical phenotype of interstitial cystitis is primarily driven by the interaction of multiple mechanisms.

### 3.1. Dysfunction of the Urinary Epithelium

The urinary epithelium is coated by a glycosaminoglycan layer comprising chondroitin sulfate, sodium hyaluronate, glycoproteins, and mucins, which collectively serve to protect the bladder [33]. Urothelial desquamation is a characteristic histological feature of interstitial cystitis. Patients with this condition often show damage to the mucosal or glycosaminoglycan layers, resulting in increased urothelial permeability to various urinary cations, such as potassium ions. This increased permeability leads to nerve and muscle depolarization, ultimately causing tissue injury. A study [34] demonstrated that bladder instillation with 0.4 M potassium chloride elicited symptoms such as urine frequency, urgency, and bladder pain in 4.5% of healthy individuals, 70% of patients with interstitial cystitis, 18% of interstitial cystitis patients undergoing heparin treatment, and 100% of individuals diagnosed with radiation cystitis. These findings may elucidate the underlying cause of bladder hypersensitivity symptoms. Additionally, studies show that interstitial cystitis with Hunner’s ulcers is characterized by pan-vesical inflammation, with Hunner’s ulcers marking the most severely exfoliated and inflamed areas [11]. Moreover, compromised differentiation of uroepithelial cells has been associated with the pathophysiology of interstitial cystitis in specific patients [35], alongside defects in keratin-18, keratin-20, and uroplakin expression [36]. Moreover, this study identified defects in the expression of differentiation-related proteins and proteoglycan core proteins—such as perlecan, biglycan, decorin, syndecan-1—in IC patients, which may correlate with factors like FGF, TGF-β, platelet-derived growth factor (PDGF), and vascular endothelial growth factor (VEGF) [36]. Protein expression abnormalities result in irregular levels of these factors, thereby disrupting downstream signaling pathways.

Recent investigations have identified pyroptosis of uroepithelial cells as a contributing factor to uroepithelial dysfunction in IC, with cytokines playing a pivotal role in this process. Pyroptosis induces rupture of the cell membrane, releasing intracellular substances including the pro-inflammatory cytokines IL-1β and IL-18 [37]. These cytokines exacerbate damage to the uroepithelial barrier, compromising the structural integrity of the bladder wall and eliciting inflammatory and painful responses. he liberated IL-1β and IL-18 stimulate the nuclear factor-κB (NF-κB) and mitogen-activated protein kinase (MAPK) signaling pathways, subsequently enhancing the expression of fibrosis-related genes, including transforming growth factor-β (TGF-β), collagen type I alpha 1 chain (COL1A1), and collagen type III alpha 1 chain (COL3A1) [38,39]. Among them, TGF-β functions as a central regulator of fibrosis by promoting fibroblast proliferation and facilitating excessive extracellular matrix (ECM) deposition, culminating in bladder wall fibrosis. Additionally, the activation of the NF-κB pathway amplifies the expression of additional pro-inflammatory mediators, such as TNF-α and IL-6, consequently exacerbating the inflammatory response and fibrotic development.

The gasdermin (GSDM) protein family shares conserved N-terminal domains that facilitate oligomerization at the cell membrane, resulting in the formation of localized death pore channels. Upon activation, caspase-1 experiences autoproteolytic cleavage into the CARD domain and the P20/P10 dimer at designated cleavage sites, subsequently cleaving gasdermin D (GSDMD) at specific residues. The proteolytic cleavage of GSDMD generates N-terminal fragments capable of associating with the cellular membrane and oligomerizing to form localized death-inducing pores [40]. The P20/P10 tetramer concurrently processes the pro-inflammatory cytokines pro-IL-1β and pro-IL-18—whose expression is upregulated via the NF-κB signaling pathway—into their mature and biologically active forms, IL-1β and IL-18. The formation of these pores induces cellular swelling as a result of osmotic water influx, facilitating the secretion of IL-1β and IL-18 through the GSDMD pores, thereby initiating a pro-inflammatory response [41]. The inflammatory mediators released during pyroptosis not only compromise the integrity of the urinary epithelial barrier but also activate sensory nerve endings within the bladder wall, contributing to heightened neurosensitivity. Empirical evidence indicates that suppression of GSDMD expression or function attenuates bladder inflammation and fibrosis [42,43], and targeting inflammatory mediators and cytokines has emerged as a promising therapeutic approach.

### 3.2. Mast Cell Proliferation and Activation

Mast cells are identified as crucial immune effector cells involved in the pathogenesis of interstitial cystitis, and their presence in the submucosal layer is a characteristic hallmark of IC/BPS with Hunner’s lesions [44]. Mast cells may be activated by a variety of mediators, including (1) stem cell factor (SCF) or nerve growth factor (NGF) released from damaged urinary epithelium; (2) bacterial and viral superantigens; (3) neuropeptides such as substance P (SP); (4) immunoglobulin aggregates; (5) acetylcholine (ACh); and (6) neuronal descending hormone [45,46,47,48,49]. Activated mast cells release vasoactive substances, inflammatory mediators, and injurious mediators through degranulation, including histamine, kinins, proteases (e.g., trypsin-like enzymes), cytokines (e.g., IL-6 and IL-8), leukotrienes, prostaglandins, and nitric oxide. Empirical evidence indicates [50] significantly increased amounts of IL-6 (*p* = 0.0054), TNF-α (*p* = 0.0064), and IL-13 (*p* = 0.0304) in the urine of IC/BPS patients exhibiting Hunner’s lesions. Additionally, increased plasma levels of IL-6, IL-8, and TNF-α have been observed in cohorts with interstitial cystitis [51]. The release of vasoactive and inflammatory mediators by mast cells provides a mechanistic basis for many clinical manifestations of interstitial cystitis. Specifically, trypsin-like proteases contribute to microvascular permeability and activate protease-activated receptors (PARs), thereby eliciting widespread inflammatory responses and neuronal hyperexcitability [52].

TNF-α released during mast cell activation promotes the activation of NF-κB, thereby intensifying inflammation within the urothelium [53]. Therapeutic intervention with the anti-TNF-α agent adalimumab has demonstrated significant clinical improvements in IC/BPS patients [54], as evidenced by reductions in the O’Leary-Sant Interstitial Cystitis Symptoms and Problems Index (*p* = 0.0002), the Interstitial Cystitis Symptoms Index (*p* = 0.0011), the Interstitial Cystitis Problems Index (*p* = 0.0002), and the pelvic pain, urgency, and frequency of urination symptom scale (*p* = 0.0017), without notable adverse effects. Vascular endothelial growth factor (VEGF), another mediator released by mast cells, promotes vasodilation, vascular proliferation, and glomeruloid alterations. Abnormal angiogenesis in the bladder has been associated with symptoms including urine frequency and bladder pain in people with IC/BPS [55]. Angiogenesis is vital for bladder tissue regeneration, as it maintains vascular supply that delivers important nutrients and oxygen through the VEGF signaling pathway, which activates the activation of Erk1/2, p38, and Akt kinases [56]. Increased expression of TNF-α, VEGF, CD31, and transforming growth factor-beta (TGF-β) have been observed in individuals with IC/BPS [57], with increased VEGF concentrations being associated with inflammatory processes in the bladder [58].

**Figure 1:**
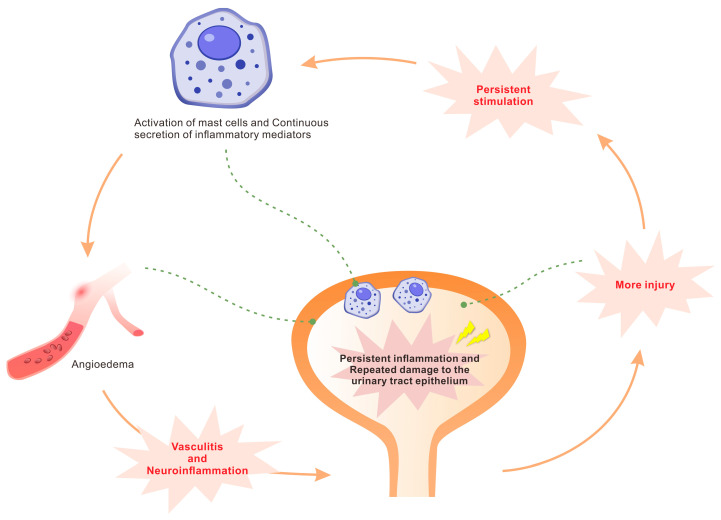
Mast cell activation contributes to the pathogenesis of interstitial cystitis.

Under normal physiological conditions, mast cells release mediators that exhibit a short half-life. In the situation of interstitial cystitis, the harmful stimulation persists for a protracted period, leading to a prolonged increase in the secretion of inflammatory mediators even after the initial stimulus has been eliminated. This prolonged mediator release induces angioedema, subsequently leading to vasculitis and neuroinflammation. The neuroinflammatory response further enhances neurotransmitter release, which in turn perpetuates mast cell activation. Consequently, this establishes a self-amplifying cycle of chronic inflammation and recurrent injury to the urinary tract epithelium (Figure 1). This pathogenic process clinically presents as symptoms associated with the lower urinary tract, like dysuria and dyspareunia, resulting from increased pain sensitivity.

### 3.3. B-Cell Clonal Expansion and Autoimmunity

Patients with interstitial cystitis (IC) often exhibit increased plasma cell aggregation, B-cell clonal expansion, and CXCR3 overexpression, which are characteristic features of this condition. Research indicates [50] that plasma cell and B-cell activation are positively correlated with the severity of interstitial cystitis. Compared to unaffected controls, bladder tissue from IC patients shows a 50-fold increase in plasma cells, a 28-fold increase in B cells, and significantly elevated urinary IL-6 levels, suggesting the involvement of these cells in disease progression. IL-6 plays a pivotal role in B-cell activation. In its presence, regulatory T cells lose their ability to suppress immune responses, potentially leading to chronic inflammation [59]. Additionally, more than 30% of IC patients exhibit light chain restriction [50], providing further evidence of B-cell clonal expansion. Research also suggests that APRIL and BAFF are key regulators of B-cell clonal expansion in IC, with significant correlations found between their gene expression levels and the extent of clonal B-cell proliferation [60]. Both APRIL and BAFF belong to the tumor necrosis factor family of ligands. BAFF promotes B-cell survival, maturation, and activation, while APRIL facilitates B-cell proliferation and plasma cell differentiation, ultimately leading to antibody production [61]. Concurrently, elevated CXCR3 expression in plasma cells within the bladder tissue of IC patients likely results from frequent B-cell clonal expansion [62]. The CXCR3 receptor is expressed in various immune cell types and primarily mediates the chemotactic migration of these cells to inflammatory sites, thereby perpetuating chronic inflammatory responses through local amplification loops.

Clonal B-cell expansion is thought to result from localized immune responses and specific B-cell clone selection, often associated with autoimmune processes. Previous studies have suggested that urinary epithelial shedding and heightened immune responses in interstitial cystitis/bladder pain syndrome (IC/BPS) are correlated with bladder tissue autoimmunity [16]. Elevated levels and concentrations of autoantibodies have been detected in both the serum and bladder tissue of individuals diagnosed with interstitial cystitis [63]. Moreover, patients suffering from systemic autoimmune disorders, including Sjögren’s syndrome, systemic lupus erythematosus, and autoimmune thyroiditis, often exhibit bladder dysfunction characterized by lower urinary tract symptoms that closely resemble those observed in patients with interstitial cystitis/bladder pain syndrome [53]. A study found that IC patients were 1.66 times more likely to be misdiagnosed with rheumatoid arthritis than the control group [64], suggesting that interstitial cystitis shares characteristics with autoimmune diseases. The presence of B-cell clonal expansion in synovial tissue of rheumatoid arthritis patients aligns with findings in the bladder tissue of IC patients [65], underscoring similarities between the two conditions. Mouse models of interstitial cystitis induced with urinary spot protein further support the autoimmune hypothesis [66].

Further evidence for the autoimmune nature of interstitial cystitis comes from the presence of Th1/Th17-polarized immune responses in the bladder, marked by significant overexpression of interferon-gamma (IFN-γ) and upregulation of IL-17A genes [67]. This is consistent with previous findings of upregulated Th1/Th17 cell-specific gene expression [68] and elevated TNF-α, IL-6, and IL-17A mRNA levels [26]. Interstitial cystitis also exhibits features commonly associated with autoimmune diseases, such as a higher prevalence in females and the presence of autoantibodies [69]. However, its classification as an autoimmune disease remains debated, primarily because specific antibodies are absent in IC patients [70]. Furthermore, bacterial or viral infections can induce B-cell clonal proliferation. Therefore, whether B-cell abnormalities are a causative factor or a response to injury in interstitial cystitis requires further investigation.

### 3.4. Neurogenic Inflammation and Pain

**Figure 2:**
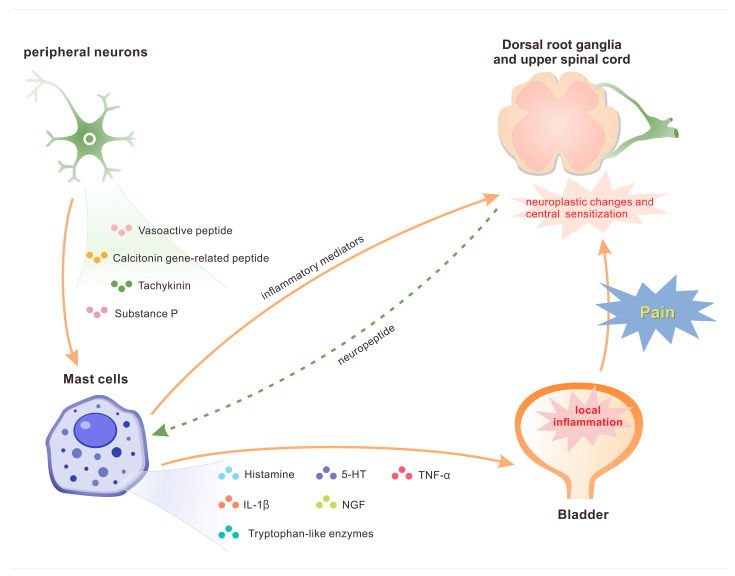
Neurogenic inflammation causes bladder pain in patients with interstitial cystitis.

The augmented release of neuropeptides from sensory and/or sympathetic nerve fibers results in sustained sensitization of afferent nerves and localized inflammatory alterations, a phenomenon termed “neurogenic inflammation”, which is primarily mediated by mast cells [71]. Neurotransmitters such as vasoactive peptide, calcitonin gene-related peptide, tachykinin, and SP, released from peripheral neurons, provoke mast cell degranulation and the subsequent secretion of pro-inflammatory mediators including 5-hydroxytryptamine, tryptophan-like enzymes, IL-1β, TNF-α, histamine, and nerve growth factor (Figure 2) [72,73]. This cascade initiates localized bladder inflammation. The released inflammatory mediators act upon afferent neurons via a positive feedback mechanism, thereby enhancing neuropeptide release and further intensifying mast cell degranulation and inflammatory responses [74]. Chronic stimulation of afferent nerves induces neuroplastic changes and central sensitization within the dorsal root ganglia and upper spinal cord, leading to the persistent symptomatology observed in IC/BPS.

Recent investigations have elucidated the involvement of Toll-like receptors (TLRs) in the pain mechanisms associated with IC/BPS. TLRs constitute a critical class of pattern recognition receptors integral to the innate immune response. Aberrant activation of TLRs in IC patients has been strongly correlated with pain and neuroinflammatory processes. One study [75] demonstrated that an increase of one standard deviation in the TLR-4-mediated inflammatory response was associated with a 1.59-fold increased chance of extrapelvic pain in IC/BPS patients (*p* = 0.019). Additionally, lower pressure pain thresholds exhibited a borderline significant association with elevated TLR-4 inflammatory responses (*p* = 0.062), while a significant correlation was observed with increased plasma IL-6 levels (*p* = 0.031). Multiple cytokines contribute to this pathophysiological process. Microglia and astrocytes exhibit expression of Toll-like receptors (TLRs), notably TLR-2 and TLR-4. The activation of TLR-4 on microglial cells induces the secretion of pro-inflammatory cytokines such as IL-6, IL-1β, and TNF-α within the spinal cord [76]. IL-17 can activate the transient receptor potential cation channel subfamily V member 4 (TRPV4) on neuronal surfaces and subsequently excite TRPV1 [77]. This activation promotes neuronal excitation, facilitating the release of pain mediators and ultimately eliciting mechanically aberrant pain sensations. Nociceptive hypersensitivity mediated by the TRPV channel family may thus exacerbate pain perception in individuals with IC/BPS.

The pathophysiological changes in IC/BPS include heightened uroepithelial permeability, uroepithelial activation, sensory nerve stimulation, infiltration of plasma cells and lymphocytes, and activation of mast cells. These activities are intimately interconnected via several synchronized positive and negative feedback loops, wherein pro-inflammatory cytokines, including IL-1β, IL-6, and TNF-α, serve as critical mediators. Table 2 provides a summary of these mechanisms and their corresponding clinical phenotypes. This self-perpetuating cycle culminates in irreversible functional and pathological changes, including fibrosis leading to reduced bladder compliance, detrusor overactivity, and nociceptive sensitization. The pathological changes collectively form the essential foundation for the chronic and repeated pain episodes and lower urinary tract symptoms typical of IC/BPS.

## 4. Conclusions and Outlook

Lifestyle modification is the primary recommended treatment for IC/BPS [78], with nearly 90% of patients reporting sensitivity to various dietary factors. Surveys indicate that citrus fruits, tomatoes, vitamin C, artificial sweeteners, coffee, tea, carbonated and alcoholic beverages, and spicy foods often exacerbate symptoms, while calcium glycerophosphate and sodium bicarbonate tend to alleviate them [79,80]. Using controlled methods, such as elimination diets, is essential for identifying dietary sensitivities and developing optimal dietary strategies. When advising patients, it is important to consider comorbid conditions and dietary influences comprehensively.

As the understanding of the inflammatory mechanisms underlying interstitial cystitis improves, various anti-inflammatory treatments are being explored. Research has shown that endoscopic low-dose triamcinolone injections produce favorable outcomes in IC patients by suppressing NF-κB and reducing the expression of IL-6, MCP-1, and IL-8, offering advantages of safety, simplicity, and minimal complications [81]. Cyclosporine A, listed as a Level V treatment in the American Urological Association guidelines for IC/BPS, has also demonstrated efficacy [82] by reducing T-cell signaling activity and decreasing IL-2 and IFN-γ synthesis. Therapeutic approaches targeting inflammatory molecules, such as cytokines, are under clinical investigation. Anti-TNF-α monoclonal antibodies, adalimumab, have shown efficacy in phase III randomized controlled trials [83]. Furthermore, some researchers suggest that phosphodiesterase 4 (PDE4) inhibitors, which simultaneously suppress TNF-α and IL-6, may have therapeutic value for IC [59], though this remains to be validated. Stem cell therapy represents a novel treatment for interstitial cystitis/bladder pain syndrome (IC/BPS). Both adult stem cells and pluripotent stem cells have shown useful in the treatment of IC/BPS. Stem cells can develop into urinary tract epithelial tissue to facilitate bladder healing and direct stem cell transplantation can mitigate inflammation in interstitial cystitis and enhance bladder function metrics [84]. The gut microbiota also have a significant impact on modulating systemic immune responses through the regulation of intestinal barrier function. Mendelian randomization analysis has revealed a positive correlation between the risk of interstitial cystitis and certain gut microbiota species, including *Butyricimonas*, *Coprococcus*, *Lactobacillales*, *Lentisphaerae*, and *Bilophila wadsworthia* [85]. Conversely, other microbial taxa, like *Desulfovibrio piger*, *Oscillibacter* *unclassified* and *Ruminococcus lactaris*, appear to exert a protective effect against the development of interstitial cystitis [85], suggesting a new therapeutic possibility.

The inflammatory mechanisms underlying IC/BPS constitute a complicated and multifaceted pathogenic process, with cytokines playing a crucial role in the initiation and course of the disease. Dysfunction of the uroepithelial barrier, considered the primary initiating event, disrupts the homeostasis of the intravesical environment, facilitating the infiltration of noxious urinary substances. This breach activates immune cells and induces the release of numerous pro-inflammatory cytokines, including IL-1β, TNF-α, and IL-6, thus initiating inflammatory reactions. Concurrently, these cytokines modulate multiple signaling pathways that sustain chronic inflammation and contribute to pain signaling and neuroinflammatory processes, clinically presenting as bladder discomfort, urine frequency, and urgency. Elucidating the specific roles of cytokines and the associated inflammatory pathways in IC/BPS is critical not only for advancing understanding of its pathogenesis but also for informing the development of novel therapeutic interventions. Future research should further explore the inflammatory mechanisms underlying IC/BPS while investigating established cytokine or pro-inflammatory pathway targets to determine their clinical value in IC/BPS treatment, thereby advancing precise diagnosis and effective management of the condition.

## Figures and Tables

**Table 2:** Clinical Phenotypes of Interstitial Cystitis and Their Underlying Mechanisms.

Clinical Phenotype	Related Mechanisms
Urothelial cell desquamation	Damage to the mucosal layer or glycosaminoglycan layer, altered urinary epithelial permeability
	Disruption of uroepithelial cell differentiation
	Pyroptosis of uroepithelial cells
Lower urinary tract symptoms such as dysuria and urgency	Mast cell activation leading to release of vasoactive substances and inflammatory mediators
	VEGF-induced vascular proliferation and glomerular-like changes
Bladder pain	Mast cell degranulation, inflammatory mediators stimulate afferent neurons, inducing neuroplastic changes and central sensitization
	Abnormal activation of Toll-like receptors
Plasma cell and B cell infiltration	Clonal expansion of B cells
	CXCR3 overexpression
	Elevated IL-6 suppresses regulatory T cells
Autoimmune phenomena	Local immune response and specific B-cell clone selection
	Th1/Th17-polarized immune response

## Data Availability

There is no original data generated or analyzed in the research process.

## References

[1] Magalhaes T.F., Baracat E.C., Doumouchtsis S.K., Haddad J.M. (2019). Biomarkers in the Diagnosis and Symptom Assessment of Patients with Bladder Pain Syndrome: A Systematic Review. Int. Urogynecol. J..

[2] Chrysanthopoulou E.L., Doumouchtsis S.K. (2014). Challenges and Current Evidence on the Management of Bladder Pain Syndrome. Neurourol. Urodyn..

[3] Lim Y., Leslie S.W., O’Rourke S. (2025). Interstitial Cystitis/Bladder Pain Syndrome.

[4] Ke Q.-S., Kuo H.-C. (2015). Pathophysiology of Interstitial Cystitis/Bladder Pain Syndrome. Tzu Chi Med. J..

[5] Jiang Y.-H., Peng C.-H., Liu H.-T., Kuo H.-C. (2013). Increased Pro-Inflammatory Cytokines, C-Reactive Protein and Nerve Growth Factor Expressions in Serum of Patients with Interstitial Cystitis/Bladder Pain Syndrome. PLoS ONE.

[6] Chennamsetty A., Khourdaji I., Goike J., Killinger K.A., Girdler B., Peters K.M. (2015). Electrosurgical Management of Hunner Ulcers in a Referral Center’s Interstitial Cystitis Population. Urology.

[7] Peters K.M., Killinger K.A., Mounayer M.H., Boura J.A. (2011). Are Ulcerative and Nonulcerative Interstitial Cystitis/Painful Bladder Syndrome 2 Distinct Diseases? A Study of Coexisting Conditions. Urology.

[8] Warren J.W., Howard F.M., Cross R.K., Good J.L., Weissman M.M., Wesselmann U., Langenberg P., Greenberg P., Clauw D.J. (2009). Antecedent Nonbladder Syndromes in Case-Control Study of Interstitial Cystitis/Painful Bladder Syndrome. Urology.

[9] Warren J.W. (2014). Bladder Pain Syndrome/Interstitial Cystitis as a Functional Somatic Syndrome. J. Psychosom. Res..

[10] Logadottir Y., Delbro D., Lindholm C., Fall M., Peeker R. (2014). Inflammation Characteristics in Bladder Pain Syndrome ESSIC Type 3C/Classic Interstitial Cystitis. Int. J. Urol..

[11] Akiyama Y., Homma Y., Maeda D. (2019). Pathology and Terminology of Interstitial Cystitis/Bladder Pain Syndrome: A Review. Histol. Histopathol..

[12] Maeda D., Akiyama Y., Morikawa T., Kunita A., Ota Y., Katoh H., Niimi A., Nomiya A., Ishikawa S., Goto A. (2015). Hunner-Type (Classic) Interstitial Cystitis: A Distinct Inflammatory Disorder Characterized by Pancystitis, with Frequent Expansion of Clonal B-Cells and Epithelial Denudation. PLoS ONE.

[13] Akiyama Y., Hanno P. (2019). Phenotyping of Interstitial Cystitis/Bladder Pain Syndrome. Int. J. Urol..

[14] Logadottir Y., Fall M., Kåbjörn-Gustafsson C., Peeker R. (2012). Clinical Characteristics Differ Considerably between Phenotypes of Bladder Pain Syndrome/Interstitial Cystitis. Scand. J. Urol. Nephrol..

[15] Homma Y., Akiyama Y., Tomoe H., Furuta A., Ueda T., Maeda D., Lin A.T., Kuo H.-C., Lee M.-H., Oh S.-J. (2020). Clinical Guidelines for Interstitial Cystitis/Bladder Pain Syndrome. Int. J. Urol..

[16] Akiyama Y., Luo Y., Hanno P.M., Maeda D., Homma Y. (2020). Interstitial Cystitis/Bladder Pain Syndrome: The Evolving Landscape, Animal Models and Future Perspectives. Int. J. Urol..

[17] Yu J., Lee C.U., Lee K.-S., Ko K.J. (2023). Optimal Endoscopic Treatment and Partial Cystectomy with or without Bladder Augmentation for Hunner-Type Interstitial Cystitis. LUTS Low. Urin. Tract. Symptoms.

[18] Derisavifard S., Moldwin R. (2020). Surgical Management of Interstitial Cystitis/Bladder Pain Syndrome. Female Pelvic Surgery.

[19] Hannoodee S., Nasuruddin D.N. (2025). Acute Inflammatory Response.

[20] Lala V., Leslie S.W., Minter D.A. (2025). Acute Cystitis.

[21] Pahwa R., Goyal A., Jialal I. (2025). Chronic Inflammation.

[22] Grover S., Srivastava A., Lee R., Tewari A.K., Te A.E. (2011). Role of Inflammation in Bladder Function and Interstitial Cystitis. Ther. Adv. Urol..

[23] Schrepf A., O’Donnell M., Luo Y., Bradley C.S., Kreder K., Lutgendorf S. (2014). Inflammation and Inflammatory Control in Interstitial Cystitis/Bladder Pain Syndrome: Associations with Painful Symptoms. Pain.

[24] Jiang Y.-H., Jhang J.-F., Hsu Y.-H., Kuo H.-C. (2022). Usefulness of Urinary Biomarkers for Assessing Bladder Condition and Histopathology in Patients with Interstitial Cystitis/Bladder Pain Syndrome. Int. J. Mol. Sci..

[25] Corcoran A.T., Yoshimura N., Tyagi V., Jacobs B., Leng W., Tyagi P. (2013). Mapping the Cytokine Profile of Painful Bladder Syndrome/Interstitial Cystitis in Human Bladder and Urine Specimens. World J. Urol..

[26] Logadottir Y., Delbro D., Fall M., Gjertsson I., Jirholt P., Lindholm C., Peeker R. (2014). Cytokine Expression in Patients with Bladder Pain Syndrome/Interstitial Cystitis ESSIC Type 3C. J. Urol..

[27] Furuta A., Yamamoto T., Suzuki Y., Gotoh M., Egawa S., Yoshimura N. (2018). Comparison of Inflammatory Urine Markers in Patients with Interstitial Cystitis and Overactive Bladder. Int. Urogynecol. J..

[28] Shah A.M., Vodovotz Y., Yoshimura N., Chermansky C.J., Fitzgerald J., Tyagi P. (2023). Temporally Complex Inflammatory Networks in an Animal Model Reveal Signatures for Interstitial Cystitis and Bladder Pain Syndrome Phenotype. Neurourol. Urodyn..

[29] Jhang J.-F., Hsu Y.-H., Kuo H.-C. (2015). Possible Pathophysiology of Ketamine-Related Cystitis and Associated Treatment Strategies. Int. J. Urol..

[30] Jiang Y.-H., Jhang J.-F., Kuo H.-C. (2022). Can We Use Urinary Cytokine/Chemokine Analysis in Discriminating Ulcer-Type Interstitial Cystitis/Bladder Pain Syndrome?. Diagnostics.

[31] Niimi A., Igawa Y., Aizawa N., Honma T., Nomiya A., Akiyama Y., Kamei J., Fujimura T., Fukuhara H., Homma Y. (2018). Diagnostic Value of Urinary CXCL10 as a Biomarker for Predicting Hunner Type Interstitial Cystitis. Neurourol. Urodyn..

[32] Lamale L.M., Lutgendorf S.K., Zimmerman M.B., Kreder K.J. (2006). Interleukin-6, Histamine, and Methylhistamine as Diagnostic Markers for Interstitial Cystitis. Urology.

[33] Parsons C.L. (2007). The Role of the Urinary Epithelium in the Pathogenesis of Interstitial Cystitis/Prostatitis/Urethritis. Urology.

[34] Soler R., Bruschini H., Freire M.P., Alves M.T., Srougi M., Ortiz V. (2008). Urine Is Necessary to Provoke Bladder Inflammation in Protamine Sulfate Induced Urothelial Injury. J. Urol..

[35] Southgate J., Varley C.L., Garthwaite M.A.E., Hinley J., Marsh F., Stahlschmidt J., Trejdosiewicz L.K., Eardley I. (2007). Differentiation Potential of Urothelium from Patients with Benign Bladder Dysfunction. BJU Int..

[36] Hauser P.J., Dozmorov M., Bane B.L., Slobodov G., Culkin D.J., Hurst R.E. (2008). Abnormal Expression of Differentiation-Related Proteins and Proteoglycan Core Proteins in the Urothelium of Interstitial Cystitis Patients. J. Urol..

[37] Hsu S.-K., Li C.-Y., Lin I.-L., Syue W.-J., Chen Y.-F., Cheng K.-C., Teng Y.-N., Lin Y.-H., Yen C.-H., Chiu C.-C. (2021). Inflammation-Related Pyroptosis, a Novel Programmed Cell Death Pathway, and Its Crosstalk with Immune Therapy in Cancer Treatment. Theranostics.

[38] Broz P. (2025). Pyroptosis: Molecular Mechanisms and Roles in Disease. Cell Res..

[39] Song Z., Gong Q., Guo J. (2021). Pyroptosis: Mechanisms and Links with Fibrosis. Cells.

[40] Wang K., Sun Q., Zhong X., Zeng M., Zeng H., Shi X., Li Z., Wang Y., Zhao Q., Shao F. (2020). Structural Mechanism for GSDMD Targeting by Autoprocessed Caspases in Pyroptosis. Cell.

[41] Jorgensen I., Rayamajhi M., Miao E.A. (2017). Programmed Cell Death as a Defence against Infection. Nat. Rev. Immunol..

[42] Osman N.I., Bratt D.G., Downey A.P., Esperto F., Inman R.D., Chapple C.R. (2021). A Systematic Review of Surgical Interventions for the Treatment of Bladder Pain Syndrome/Interstitial Cystitis. Eur. Urol. Focus..

[43] Wang X., Yin H., Fan L., Zhou Y., Tang X., Fei X., Tang H., Peng J., Zhang J., Xue Y. (2021). Shionone Alleviates NLRP3 Inflammasome Mediated Pyroptosis in Interstitial Cystitis Injury. Int. Immunopharmacol..

[44] Gamper M., Regauer S., Welter J., Eberhard J., Viereck V. (2015). Are Mast Cells Still Good Biomarkers for Bladder Pain Syndrome/Interstitial Cystitis?. J. Urol..

[45] Theoharides T.C., Kempuraj D., Sant G.R. (2001). Mast Cell Involvement in Interstitial Cystitis: A Review of Human and Experimental Evidence. Urology.

[46] Lowe E.M., Anand P., Terenghi G., Williams-Chestnut R.E., Sinicropi D.V., Osborne J.L. (1997). Increased Nerve Growth Factor Levels in the Urinary Bladder of Women with Idiopathic Sensory Urgency and Interstitial Cystitis. Br. J. Urol..

[47] Pang X., Sant G., Theoharides T.C. (1998). Altered Expression of Bladder Mast Cell Growth Factor Receptor (c-Kit) in Interstitial Cystitis. Urology.

[48] Peeker R., Enerbäck L., Fall M., Aldenborg F. (2000). Recruitment, Distribution and Phenotypes of Mast Cells in Interstitial Cystitis. J. Urol..

[49] Dudeck J., Kotrba J., Immler R., Hoffmann A., Voss M., Alexaki V.I., Morton L., Jahn S.R., Katsoulis-Dimitriou K., Winzer S. (2021). Directional Mast Cell Degranulation of Tumor Necrosis Factor into Blood Vessels Primes Neutrophil Extravasation. Immunity.

[50] Moldwin R.M., Nursey V., Yaskiv O., Dalvi S., Macdonald E.J., Funaro M., Zhang C., DeGouveia W., Ruzimovsky M., Rilo H.R. (2022). Immune Cell Profiles of Patients with Interstitial Cystitis/Bladder Pain Syndrome. J. Transl. Med..

[51] Jiang C., Xu M., Zhu J., Yang D., Xue B. (2022). CircTHBS1 Facilitates the Progression of Interstitial Cystitis Depending on the Regulation of miR-139-5p/MFN2 Axis. Drug Dev. Res..

[52] Varricchi G., Rossi F.W., Galdiero M.R., Granata F., Criscuolo G., Spadaro G., de Paulis A., Marone G. (2019). Physiological Roles of Mast Cells: Collegium Internationale Allergologicum Update 2019. Int. Arch. Allergy Immunol..

[53] Mostafa M.M., Kamel M., Kamel M., Mahdy A. (2024). Interstitial Cystitis/Bladder Pain Syndrome: Role of Bladder Inflammation in Bladder Function. Curr. Bladder Dysfunct. Rep..

[54] Bosch P.C. (2014). A Randomized, Double-Blind, Placebo Controlled Trial of Adalimumab for Interstitial Cystitis/Bladder Pain Syndrome. J. Urol..

[55] Furuta A., Suzuki Y., Igarashi T., Koike Y., Kimura T., Egawa S., Yoshimura N. (2019). Angiogenesis in Bladder Tissues Is Strongly Correlated with Urinary Frequency and Bladder Pain in Patients with Interstitial Cystitis/Bladder Pain Syndrome. Int. J. Urol..

[56] Gee E., Milkiewicz M., Haas T.L. (2010). P38 MAPK Activity Is Stimulated by Vascular Endothelial Growth Factor Receptor 2 Activation and Is Essential for Shear Stress-Induced Angiogenesis. J. Cell. Physiol..

[57] Lin H.-Y., Lu J.-H., Chuang S.-M., Chueh K.-S., Juan T.-J., Liu Y.-C., Juan Y.-S. (2021). Urinary Biomarkers in Interstitial Cystitis/Bladder Pain Syndrome and Its Impact on Therapeutic Outcome. Diagnostics.

[58] Peng C.-H., Jhang J.-F., Shie J.-H., Kuo H.-C. (2013). Down Regulation of Vascular Endothelial Growth Factor Is Associated with Decreased Inflammation after Intravesical OnabotulinumtoxinA Injections Combined with Hydrodistention for Patients with Interstitial Cystitis--Clinical Results and Immunohistochemistry Analysis. Urology.

[59] Su F., Zhang W., Meng L., Zhang W., Liu X., Liu X., Chen M., Zhang Y., Xiao F. (2022). Multimodal Single-Cell Analyses Outline the Immune Microenvironment and Therapeutic Effectors of Interstitial Cystitis/Bladder Pain Syndrome. Adv. Sci..

[60] Horie M., Akiyama Y., Katoh H., Taguchi S., Nakamura M., Mizuguchi K., Ito Y., Matsushita T., Ushiku T., Ishikawa S. (2024). APRIL/BAFF Upregulation Is Associated with Clonal B-Cell Expansion in Hunner-Type Interstitial Cystitis. J. Pathol..

[61] Mackay F., Schneider P., Rennert P., Browning J. (2003). BAFF AND APRIL: A Tutorial on B Cell Survival. Annu. Rev. Immunol..

[62] Akiyama Y., Morikawa T., Maeda D., Shintani Y., Niimi A., Nomiya A., Nakayama A., Igawa Y., Fukayama M., Homma Y. (2016). Increased CXCR3 Expression of Infiltrating Plasma Cells in Hunner Type Interstitial Cystitis. Sci. Rep..

[63] Ochs R.L., Stein T.W., Peebles C.L., Gittes R.F., Tan E.M. (1994). Autoantibodies in Interstitial Cystitis. J. Urol..

[64] Keller J.J., Liu S.-P., Lin H.-C. (2013). A Case–Control Study on the Association between Rheumatoid Arthritis and Bladder Pain Syndrome/Interstitial Cystitis. Neurourol. Urodyn..

[65] Doorenspleet M.E., Klarenbeek P.L., de Hair M.J.H., van Schaik B.D.C., Esveldt R.E.E., van Kampen A.H.C., Gerlag D.M., Musters A., Baas F., Tak P.P. (2014). Rheumatoid Arthritis Synovial Tissue Harbours Dominant B-Cell and Plasma-Cell Clones Associated with Autoreactivity. Ann. Rheum. Dis..

[66] Altuntas C.Z., Daneshgari F., Sakalar C., Goksoy E., Gulen M.F., Kavran M., Qin J., Li X., Tuohy V.K. (2012). Autoimmunity to Uroplakin II Causes Cystitis in Mice: A Novel Model of Interstitial Cystitis. Eur. Urol..

[67] Akiyama Y., Harada K., Miyakawa J., Kreder K.J., O’Donnell M.A., Daichi M., Katoh H., Hori M., Owari K., Futami K. (2023). Th1/17 Polarization and Potential Treatment by an Anti-Interferon-γ DNA Aptamer in Hunner-Type Interstitial Cystitis. iScience.

[68] Akiyama Y., Miyakawa J., Horie M., Saito T., Minagawa T., Ushiku T., Goto A., Kume H., Homma Y., Luo Y. (2025). Molecular Characterization of Chronic Inflammatory Diseases of the Urinary Bladder Based on Next-Generation RNA Sequencing and Digital Image Analysis. Sci. Rep..

[69] van de Merwe J.P. (2007). Interstitial Cystitis and Systemic Autoimmune Diseases. Nat. Rev. Urol..

[70] Fallon J., Stern I.T., Laurent M., Birder L., Moldwin R.M., Stern J.N.H. (2023). The Immune System in Interstitial Cystitis/Bladder Pain Syndrome and Therapeutic Agents. Continence.

[71] Matsuda M., Huh Y., Ji R.-R. (2019). Roles of Inflammation, Neurogenic Inflammation, and Neuroinflammation in Pain. J. Anesth..

[72] Amaya F., Izumi Y., Matsuda M., Sasaki M. (2013). Tissue Injury and Related Mediators of Pain Exacerbation. Curr. Neuropharmacol..

[73] Ji R.-R., Xu Z.-Z., Gao Y.-J. (2014). Emerging Targets in Neuroinflammation-Driven Chronic Pain. Nat. Rev. Drug Discov..

[74] Ito A., Hagiyama M., Oonuma J. (2008). Nerve-Mast Cell and Smooth Muscle-Mast Cell Interaction Mediated by Cell Adhesion Molecule-1, CADM1. J. Smooth Muscle Res..

[75] Schrepf A., Bradley C.S., O’Donnell M., Luo Y., Harte S.E., Kreder K., Lutgendorf S. (2015). Toll-like Receptor 4 and Comorbid Pain in Interstitial Cystitis/Bladder Pain Syndrome: A Multidisciplinary Approach to the Study of Chronic Pelvic Pain Research Network Study. Brain Behav. Immun..

[76] Milligan E.D., Watkins L.R. (2009). Pathological and Protective Roles of Glia in Chronic Pain. Nat. Rev. Neurosci..

[77] Luo X., Chen O., Wang Z., Bang S., Ji J., Lee S.H., Huh Y., Furutani K., He Q., Tao X. (2021). IL-23/IL-17A/TRPV1 Axis Produces Mechanical Pain via Macrophage-Sensory Neuron Crosstalk in Female Mice. Neuron.

[78] Imamura M., Scott N.W., Wallace S.A., Ogah J.A., Ford A.A., Dubos Y.A., Brazzelli M. (2020). Interventions for Treating People with Symptoms of Bladder Pain Syndrome: A Network Meta-analysis. Cochrane Database Syst. Rev..

[79] Friedlander J.I., Shorter B., Moldwin R.M. (2012). Diet and Its Role in Interstitial Cystitis/Bladder Pain Syndrome (IC/BPS) and Comorbid Conditions. BJU Int..

[80] Bassaly R., Downes K., Hart S. (2011). Dietary Consumption Triggers in Interstitial Cystitis/Bladder Pain Syndrome Patients. Female Pelvic Med. Reconstr. Surg..

[81] Funaro M.G., King A.N., Stern J.N.H., Moldwin R.M., Bahlani S. (2018). Endoscopic Injection of Low Dose Triamcinolone: A Simple, Minimally Invasive, and Effective Therapy for Interstitial Cystitis with Hunner Lesions. Urology.

[82] Forrest J.B., Payne C.K., Erickson D.R. (2012). Cyclosporine A for Refractory Interstitial Cystitis/Bladder Pain Syndrome: Experience of 3 Tertiary Centers. J. Urol..

[83] Li J., Yi X., Ai J. (2022). Broaden Horizons: The Advancement of Interstitial Cystitis/Bladder Pain Syndrome. Int. J. Mol. Sci..

[84] Abdal Dayem A., Kim K., Lee S.B., Kim A., Cho S.-G. (2020). Application of Adult and Pluripotent Stem Cells in Interstitial Cystitis/Bladder Pain Syndrome Therapy: Methods and Perspectives. J. Clin. Med..

[85] Fu C., Zhao Y., Zhou X., Lv J., Jin S., Zhou Y., Liu F., Feng N. (2024). Gut Microbiota and Interstitial Cystitis: Exploring the Gut-Bladder Axis through Mendelian Randomization, Biological Annotation and Bulk RNA Sequencing. Front. Immunol..

